# Navigating Conflicts of Duty in Contexts of Compassionate Care: Utilitarian Reasoning as a Practice‐Oriented Framework

**DOI:** 10.1111/nin.70138

**Published:** 2026-07-21

**Authors:** Ricardo Ayala, Daniela Castillo, Pierre Pariseau‐Legault

**Affiliations:** ^1^ Faculty of Health & Social Sciences Universidad de las Américas Santiago de Chile Chile; ^2^ Faculty of Nursing Sciences University of Montreal Montreal Canada; ^3^ Universidad de Chile Nursing School Santiago de Chile Chile; ^4^ Université du Québec en Outaouais Canada

**Keywords:** clinical ethics, compassion, compassionate care, moral reasoning, nursing ethics, utilitarianism

## Abstract

Compassionate nursing practice frequently requires nurses to navigate competing obligations, conflicting patient needs and constrained resources. While compassion motivates responsiveness to suffering, it does not always provide sufficient guidance for determining how such conflicts should be resolved. We argue that contemporary forms of utilitarianism may offer a useful framework for structuring compassionate practice in nursing within ethically pluralistic clinical environments. Drawing on contemporary utilitarian thought, particularly preference utilitarianism, the paper examines how attentiveness to suffering, preservation of dignity and responsiveness to patient vulnerability may be reframed around ethical deliberation under competing demands. Through clinical examples, we explore how compassionate nursing practice often involves the weighing of competing preferences, the prioritisation of morally significant needs and the allocation of finite professional attention. We argue that preference utilitarianism can fruitfully provide a structured ethical resource for evaluating actions according to their capacity to reduce and prevent suffering while respecting individual preferences and circumstances. The paper does not propose a foundational moral theory for nursing. Rather, it argues that preference utilitarianism can function as a complementary ethical resource alongside care ethics, virtue‐based approaches and other relational perspectives, particularly in situations where nurses must navigate conflicting ethical duties. It therefore demonstrates how compassionate actions can be morally justified and systematically assessed, supporting a move from isolated acts of care towards more sustained and context‐sensitive ethical practices within healthcare systems.

## 1 Introduction

Nurses routinely encounter situations in which multiple patients suffer simultaneously, resources are limited and not all morally relevant needs can be met. Such situations create conflicts of duty that require both prioritisation and justification. Compassion remains an indispensable moral orientation within nursing practice and caregiving (Schantz 2007; McCaffrey and McConnell 2015; Raustøl and Tveit 2022). Its association with the historical identities constructed around the profession is so strong that it can obscure nurses' knowledge and competencies (Foth and Nazon 2022). At the same time, compassion has been criticised in regard to its ethicality when expressions of care risk undermining patient autonomy or reinforcing dependency (Fountouki et al. 2020; Reeves 2017). And yet, despite commonplace narratives of compassion in contemporary care systems and health policies, compassion alone may not always determine how nurses ought to act in settings involving the myriad clinical duties that can potentially collide. We argue in this paper that utilitarian philosophy can provide a structured ethical resource for navigating such conflicts within an ethically pluralistic framework.

In constructing its ethical foundation, nursing has drawn upon multiple philosophical traditions. Kantian deontology, with its emphasis on duty and moral law, has provided an enduring framework, offering consistency and clarity (Kant 1998). Yet, its rational and rule‐bound nature is sometimes viewed as distant from the relational and emotional dimensions of care. Earlier sentimentalist thinkers, particularly David Hume, argued that moral judgement depends on reason but also on sympathy and fellow‐feeling, which highlights the affective dimensions of ethical life. Compassion has likewise occupied a central place in more recent moral philosophy. Compassion has therefore been proposed as a necessary complement, restoring attention to the human connection between individuals (Sinclair, Norris, et al. 2016; Bond et al. 2024). The challenge of integrating compassionate responsiveness with practical ethical judgement has deep philosophical roots, extending from Aristotle's concept of *phronesis* to Cortina (2007) notion of cordial reason. When insufficiently reflected upon, compassion may indeed risk becoming instrumental—a means to achieve moral satisfaction for the caregiver rather than genuine relief for the cared‐for. This is no trivial concern. It highlights the potential for caregiving to become emotionally superficial or merely ‘transactional’ rather than transformative (Clinton 2025; Pattison and Corser 2023).

The central question, then, is whether contemporary utilitarianism can offer a useful ethical framework for navigating conflicting clinical duties and competing needs within clinical practice. Rather than proposing utilitarianism as an overarching moral theory for nursing, this paper examines how a preference‐utilitarian perspective can illuminate and structure aspects of compassionate clinical reasoning alongside existing ethical frameworks. This framework, grounded in the reduction and prevention of suffering, offers analytical guidance and, by the same token, contributes to nursing's ethically pluralistic reasoning. Beneath this goal lies the premise that compassion is understood as a response that requires ethical deliberation when nurses encounter competing obligations, conflicting needs and resource constraints. While clarifying this conception can help refine theory, it strengthens nursing's capacity to respond to conflicts of duty with consistency and moral clarity.

While this analysis situates compassion within a contemporary utilitarian framework, it is important to acknowledge its proximity and tension with care ethics. Ethicists of care (Gilligan 1993, 2018) conceive responsiveness to needs not as an instrumental means to maximise well‐being, but as a relational practice grounded in attentiveness, responsibility and responsiveness. In contrast, this discussion argues that a utilitarian reading of compassion, if contemporary and contextualised, can coexist with such relational ethics rather than oppose them. Within nursing, compassion often functions as an ‘enveloping’ ethical orientation within specific contexts, shaping the delivery of concrete interventions in situations of suffering. It guides action—the ethical adequacy of compassion should thus be judged not by theoretical purity but by practical coherence within clinical realities. Nurses necessarily draw upon what makes sense in practice—some degree of clinical pragmatism remains essential. A utilitarian interpretation of compassion, then, need not be seen as reductive, but as articulating the broader social and institutional aims of compassionate care, while remaining sensitive to the relational and moral dimensions emphasised by care theories.

In clinical practice, such dimensions of compassion frequently arise in situations where multiple legitimate obligations cannot be fully satisfied simultaneously. In this sense, preference utilitarianism is explored as a framework for navigating competing needs, and clinically complex ethical situations, thereby offering a coherent and practically useful contribution to modern nursing ethics.

The paper proceeds as follows. Section 2 examines the conceptual foundations of compassion, its expression in practice and the ethical limitations that arise when nurses encounter competing needs and obligations. Section 3 then considers a contemporary utilitarian framework and examines whether it can provide a coherent ethical grounding for compassion within nursing by applying a structured ethical analysis. Two hypothetical scenarios illustrate its application. Finally, the paper reflects on the implications of this account for practice, with particular attention to the need to integrate utilitarian reasoning into inherently pluralistic clinical ethics.

## 2 Key Concepts

Since antiquity, philosophers have understood ethical life as demanding both concern for others and practical wisdom in responding to their needs. In the Aristotelian tradition, moral excellence depends not just upon good intentions but upon *phronesis* (practical wisdom), the ability to perceive what is morally significant in particular circumstances and to act appropriately. More recently, Adela Cortina has argued that ethical judgement emerges through the integration of rational deliberation and affective responsiveness, a synthesis she describes as *razón cordial* (cordial reason). Although 24 centuries apart, both philosophers converge in viewing ethical judgement as requiring both rational deliberation and sensitivity to human vulnerability. This insight is particularly relevant to current healthcare contexts, where compassion may reveal suffering but does not always determine how competing obligations should be prioritised. Compassionate competence is not to be understood as a soft skill that operates independently of clinical expertise; it constitutes a mechanism through which knowledge and judgement are translated into care that is meaningful and responsive to individual patient needs. Ethical caregiving therefore requires not only responsiveness to vulnerability but also frameworks capable of guiding deliberation when duties collide.

At present, compassion occupies a prominent place within nursing, yet it remains conceptually contested. It is frequently conflated with related concepts such as empathy, person‐centred care and humanised care, creating conceptual ambiguity regarding its distinct ethical role (Portoghese et al. 2020; Sinclair, Norris, et al. 2016; Gilbert 2017). Although definitions vary, contemporary scholarship broadly converges on compassion as a response to suffering that seeks to preserve dignity and alleviate distress (Chochinov 2007; Sinclair, McClement, et al. 2016; Pfaff and Markaki 2017; Sinclair et al. 2017; Malenfant et al. 2022).

Nursing practice is inherently intertwined with suffering, making compassion particularly salient within clinical care (de Zulueta Zuleta 2015; Gilbert 2019; Malenfant et al. 2022; Ferrell and Rosa 2023). Compassion is commonly understood as a sensitivity to suffering accompanied by a commitment to alleviate and prevent it (Gilbert 2014, 2019), and suffering itself extends beyond physical pain to encompass emotional, social, existential and spiritual dimensions (Cassell 2004; Egnew 2009; de Zulueta Zuleta 2015; Kanov 2021). Experiences such as fear, loneliness and grief illustrate how suffering may arise in diverse forms while retaining a common capacity to threaten well‐being and personhood.

Across the literature, several interrelated attributes consistently characterise compassion. These include attentiveness to suffering (Gilbert 2014, 2019; Sinclair, Norris, et al. 2016; Cole‐King and Gilbert 2014), empathic understanding (Klimecki et al. 2014; Dewar et al. 2014; Malenfant et al. 2022), moral motivation directed towards alleviating suffering (Goetz et al. 2010; Chochinov 2007; Gilbert 2019), relational presence (Dewar et al. 2011; Dewar et al. 2014; Menage et al. 2020), respect for dignity and personhood (Corbin 2008; Dewar et al. 2011; Sinclair et al. 2017) and concrete expressions of care through both kindness and professional competence (Maben et al. 2018; Smith 2012).

Compassion nevertheless remains distinct from neighbouring concepts. Empathy facilitates emotional understanding but does not necessarily entail action (Jinpa 2010; Seppälä et al. 2017). Sympathy may express concern while maintaining distance, whereas pity is often experienced as condescending and undermining of dignity (Chochinov 2007; Sinclair et al. 2017). Likewise, while person‐centred care, caring and holistic care overlap substantially with compassion, they constitute broader frameworks not exclusively oriented towards suffering (Watson 1988; Freeman 2015; McCormack and McCance 2010; Dewar et al. 2016; Carvajal et al. 2019).

Taken together, compassion may be understood as an active, relational and ethically directed response to suffering that integrates emotional sensitivity with purposeful action.

### 2.1 Compassion in Practice: Conditions, Expressions and Ethical Limits

While important, compassion does not arise solely from individual disposition. Rather, its expression depends upon the interaction of personal, relational, organisational and cultural factors. Individual influences include values, emotional awareness, life experience and reflexive capacity (Christiansen et al. 2015; Gilbert 2017; Hochschild 1983; Smith 2012). Relationally, compassion depends upon communication, trust, psychological safety and authentic human presence (Edmondson 2019; Gilbert 2020).

Supportive and psychologically safe environments tend to foster compassion, whereas fragmented systems, incivility and efficiency‐driven cultures may undermine it (Chen et al. 2025; Gustafsson and Hemberg 2022). Yet, organisational conditions exert considerable influence. Staffing levels, workload, leadership styles, team culture and prevailing models of care can either facilitate or constrain compassionate practice (Stenhouse et al. 2016; Tierney et al. 2019; Liberati et al. 2023). Compassion can indeed be expressed through the appropriate allocation of resources and the management of the turbulence inherent in healthcare institutions. Compassion is further shaped by wider cultural and societal influences, including beliefs about suffering, personhood and care (Gilbert 2017; Bedregal et al. 2020).

Empirical research consistently demonstrates that compassion is experienced through observable behaviours rather than abstract ideals. Not that ideals are unimportant, but it is in the interpersonal encounter where these ideals manifest. Patients recognise compassion through presence, attentiveness, responsiveness, respectful communication and actions that preserve dignity (Burnell and Agan 2013; Sinclair et al. 2017; Trzeciak et al. 2017). Its presence is associated with enhanced trust, psychological well‐being, reduced anxiety, improved patient safety and stronger care experiences (Lown et al. 2011; Tehranineshat et al. 2019; Kim and Lee 2020; Trzeciak et al. 2017). Healthcare professionals similarly report greater fulfilment and resilience when compassionate practice is supported, whereas its obstruction may contribute to burnout, compassion fatigue and moral distress (Whitehead et al. 2015; Worline and Dutton 2017; Gustafsson and Hemberg 2022; Morley and Sankary 2023).

This helps explain why compassion remains a central aspiration within nursing practice. However, care‐based approaches provide comparatively less guidance when nurses encounter conflicting duties, competing patient needs or constraints on time and resources. Such circumstances require forms of ethical reasoning capable not only of motivating action but also of supporting deliberation and justification. It is to this challenge that the present paper seeks to contribute. If compassion reveals suffering and practical wisdom helps identify what is morally salient, how should nurses decide between competing obligations when not all compassionate responses can be pursued simultaneously? In what follows, the discussion examines how preference utilitarianism may provide a structured framework for ethical deliberation in nursing practice.

## 3 Compassion Through the Lens of Utilitarianism

With compassion's conceptual dimensions established, the discussion now reconsiders it alongside contemporary utilitarian ethics. Over the two centuries following its emergence, utilitarianism acquired a rather contentious reputation in bioethics,^1^ often criticised for its perceived failure to balance individual and collective interests (Dale 2020). Yet this reputation invites reconsideration. When viewed through the lens of compassion‐informed care, utilitarian reasoning may appear less as a cold calculus and more as a structured response to suffering. This is not to suggest that compassion should be understood as utilitarian in nature; rather, some contemporary utilitarian approaches may help articulate how competing duties can be weighed in nursing contexts marked by suffering.

### 3.1 Reframing Contemporary Utilitarianism

Although utilitarianism may be regarded as outdated and incompatible with modern professional ethics, these criticisms largely target its *classical* formulation. Subsequent developments, however, have produced more nuanced versions that seek to reconcile individual moral concern with broader considerations of justice and policy (Liu 2023). It is within these contemporary reinterpretations that compassion‐informed care can be situated as a morally responsive extension rather than a philosophical departure.

Although often portrayed as in tension with holistic and humanistic ethics (Henderson 2002) and criticised for an excessive focus on instrumental skills (Scott 2008), contemporary utilitarianism remains present—and at times even recognised—within nursing science. It appears not only in clinical education (Berndt 2010), nursing management (Chaudhary et al. 2024) and disaster response (Caggianelli et al. 2025) but also in the ethical reasoning that underpins professional judgement (Berndt 2010). Likewise, utilitarianism is embedded in the organisation of day‐to‐day clinical work—particularly in prioritisation—where superordinate values structure decisions related to patient safety, thereby reconnecting everyday practices with their ethical foundations (Wright et al. 2021).

Utilitarian reasoning may therefore function as an ‘unofficial ethical theory’ (DesJardins 2006; Nilsen 2010), guiding professional decision‐making widely, albeit often implicitly. It likewise appears as a dominant conception of justice in medical ethics and public health policy, where resource allocation is typically justified through utilitarian principles (Rhodes 2025). Rather than operating as a purely calculative doctrine, contemporary utilitarianism is better understood as interacting with other ethical principles, most notably egalitarian concerns (Stein 2012).

Consequently, contemporary utilitarianism can be seen as a widely operative—though not exclusive—framework in professional ethics, insofar as it helps justify policies and practices aimed at morally reasonable outcomes. This becomes particularly relevant in nursing because conflicts of duty frequently require choices between multiple morally defensible actions rather than between right and wrong actions. This is most visible under conditions of scarcity. For instance, triage protocols in emergency settings reflect this reasoning by directing limited resources towards interventions most likely to produce the greatest overall reduction of suffering.

### 3.2 Compassion and Moral Utility

Moving beyond classical utilitarianism's focus on aggregate outcomes, this article proposes a re‐reading of contemporary utilitarianism as a framework for understanding compassion, as a structure for guiding (and assessing) morally relevant responses to suffering. This distinction matters. It becomes particularly relevant in models of care, where the literature rarely frames utilitarianism explicitly in terms of compassion—perhaps because utility is often (mis)interpreted as being in tension with humanistic moral theories. It is particularly relevant in contexts where responses to suffering must be both immediate and justifiable. While classical utilitarianism does acknowledge the importance of reducing suffering—Bentham^2^ notably extended moral concern beyond rational human beings (French 2008)—its primary focus remains the maximisation of overall happiness, or the greatest good for the greatest number of individuals (Liu 2023). In doing so, individuals may be treated as interchangeable units within a broader calculation of pleasure and pain. Contemporary reformulations, however, particularly negative utilitarianism and preference utilitarianism,^3^ shift the focus towards the qualitative significance of suffering and the moral relevance of individual experience. This orientation renders their application compatible with compassionate reasoning. And more attentive to lived experience.

In particular, negative utilitarianism and preference utilitarianism reposition ethical concern by foregrounding the reduction of suffering and the consideration of individual interests. Negative utilitarianism prioritises the reduction of suffering over the maximisation of pleasure, aligning closely with compassionate reasoning. Similarly, Peter Singer's preference utilitarianism emphasises that what counts in calculating the expected utility of a decision is the preferences of the sentient beings affected by it. Reformulated in a care‐oriented context, this implies alleviating suffering by attending to the preferences and well‐being of those involved. Approached this way, preference utilitarianism is certainly compatible with compassion and autonomous decision‐making in the nursing profession.

The relevance of preference utilitarianism to nursing does not lie primarily in offering a theory of compassion, but in providing a method for resolving conflicts of duty when multiple compassionate responses are possible. For a compassionate reappraisal of utilitarian caregiving to be possible, its ethical commitments require reconsideration in ways that foreground responsiveness to suffering over abstract calculation. Three shifts are essential:

– First, ethical evaluation must extend beyond whether utility has been maximised to include scrutiny of the moral legitimacy of the means by which outcomes are achieved. This shift creates conceptual space for compassion as a morally relevant component of utility.
– Second, interests cannot be abstracted from the individuals who hold them: individuals must be treated as moral subjects with distinct needs, vulnerabilities and circumstances—that is, with particular and irreducible preferences. Accordingly, what matters morally is not only whether utility has been maximised in the aggregate, but whether the concrete preferences of each person affected have been genuinely taken into account in reaching that outcome.
– Third, addressing collective well‐being requires that the preferences of all those affected be considered impartially, including those least able to make their needs heard. Rather than reducing public interest to a simple aggregate of individual outcomes, this principle orients moral action towards the fair consideration of every preference at stake.

Together, these shifts reposition the reduction of suffering as a morally significant dimension of utility, and not as a detached calculation. In the context of the nurse–patient encounter, these considerations translate into everyday ethical judgements about how to respond to suffering in ways that are not only effective but also fair, proportionate and respectful of the patient as a moral subject. This raises a more difficult question. Should nurses *always* act in accordance with utilitarian principles (i.e., reducing suffering regardless of the circumstances)? And how far should utility be maximised in a given scenario (i.e., what level of suffering reduction is reasonable or sufficient)? This context‐sensitive reasoning helps nurses to deliberate and choose how best to proceed ethically.

Accordingly, contemporary utilitarians articulate a more nuanced formulation that seeks to prevent actions that could cause severe harm, even if they increase overall happiness. Through this theoretical evolution, the very meaning of utility itself has shifted. Utility is no longer neutral. As Singer (1993) argues, both suffering and happiness are morally significant, grounding morality among other things in the reduction of what is bad and the promotion of what is good, a formulation that positions moral life as a deliberate responsiveness to suffering rather than a mere pursuit of pleasure. This shift transforms utilitarian reasoning into a framework that is more attentive to the lived experiences of others.

Contemporary utilitarianism has sought to address longstanding criticisms concerning rights, justice and equality while retaining the broader commitment to evaluating actions according to their consequences. Rather than requiring that every individual act directly maximise utility, contemporary approaches increasingly recognise the role of moral rules, institutions and social practices in promoting ethically desirable outcomes. As Liu (2023, 6) observes:

Contemporary utilitarianism has abandoned the principle of direct utilitarianism and allowed the systems and rules to play greater roles, thus avoiding conflicts between utilitarian calculation and moral common senses. Contemporary utilitarianism has realized the integration of utility maximization and individual rights within the theory, ensuring the independence and integrity of individuals.

Moreover, despite ongoing tensions between individual and collective interests, different strands of utilitarian philosophy show that contemporary utilitarianism is plural and internally differentiated, but unified by a shared commitment to evaluating moral and political questions in terms of their consequences for human welfare. Preference utilitarianism is one of the main contemporary strands defined by a revised understanding of what ‘utility’ means. Instead of identifying utility with pleasure or happiness (as in classical hedonistic utilitarianism), it understands utility in terms of the satisfaction of individuals' preferences or desires (e.g., Brandt 1979; Broome 1991; Singer 2011). In this respect, contemporary utilitarianism may be understood as an attempt to reconcile concern for overall well‐being with considerations of fairness, justice and equal moral consideration.

And so, even though the term might suggest otherwise, framing compassion within utilitarianism does not diminish its moral force. It can, in fact, operate as a structured moral framework that aligns the intent to alleviate suffering with reasoned judgement aimed at minimising harm, offering a coherent guide for ethical decision‐making in nursing practice. While preference utilitarianism offers a structured means of evaluating compassionate action, tensions remain where aggregate preference satisfaction conflicts with the dignity or inviolability of individuals. Compassion, in this light, provides the moral orientation towards reducing suffering and protecting dignity, while preference utilitarianism contributes a structured mode of ethical reasoning.

This reappraisal has direct implications for how nursing practice is understood. In their day‐to‐day work, nurses routinely engage in a form of preference‐sensitive reasoning: they weigh the likely consequences of their actions and omissions, attend to the particular needs and vulnerabilities of each patient and orient their decisions towards what is most beneficial given the patient's condition and expressed or inferred preferences. We propose that preference utilitarianism provides a structured method for adjudicating between competing clinical obligations when compassionate motivations alone do not determine priority. This view complements, rather than replaces, care ethics by grounding the emotional responsiveness of compassion in a rational and accountable commitment to alleviate suffering. Compassion, therefore, becomes accountable because decisions can be justified through explicit ethical reasoning.

### 3.3 Applications in Clinical Practice

Within nursing practice, a contemporary utilitarian framework can be particularly pertinent: the nurse–patient encounter often involves balancing human sensitivity with efficiency, individual needs with institutional constraints and immediate comfort with long‐term well‐being. These tensions are unavoidable. As becomes visible in the literature, nursing decisions rarely involve explicit philosophical deliberation (DeWolf 1989; Gallagher 2012; Goethals et al. 2010). Instead, ethical reasoning often emerges through practical judgements—through ‘what works’—shaped by context, experience and professional values (Ecarnot et al. 2017; O'Callaghan et al. 2020). Nurses routinely weigh consequences, prioritise resources and select interventions that aim at the greatest reduction of harm. This applies to both patients and communities. This illustrates how ethical practice can be both affectively grounded and outcome‐oriented, integrating emotional responsiveness with structured reasoning. Compassion alone may identify suffering, but compassion informed by utilitarian reasoning enables nurses to weigh competing demands without relinquishing sensitivity to individual suffering—holding assessment and care in deliberate tension.

Together, these observations suggest that professional care can be supported by a contemporary utilitarian ethic—one that moves deliberatively towards reducing suffering while remaining attentive to fairness, proportionality and the preferences of those affected. Despite its long philosophical lineage, utilitarianism has been relatively underexplored as a framework for meaningful nursing practice, perhaps due to its reputation for abstraction and calculation. Yet when understood in its preference form, utilitarianism offers a useful philosophically driven framework for analysis in healthcare; one that enhances the consistency, fairness and justice of care. Its value becomes practical. This alignment is meaningful even when the nurse is not explicitly guided by care ethics, since it grounds compassionate action in rational ethical principles. Nevertheless, caution remains necessary: even a compassion‐oriented utilitarianism risks becoming overly calculative if detached from relational awareness. Maintaining this balance requires the integration of reflective practice and empathic engagement, ensuring that ethical reasoning remains grounded in human experience.

Consider the following hypothetical scenario, which exemplifies a conflict of duty likely to be readily recognised by readers:

A nurse in a busy oncology ward is caring for a middle‐aged man with advanced cancer. The patient has just learned that curative treatment is no longer possible. He sits silently, avoiding eye contact, while his family waits anxiously outside. Several other patients require her attention in the same ward. The nurse notices his withdrawn posture and trembling hands. She pauses, pulls a chair close to the bedside and sits quietly with him. She listens as he expresses fear – of death, of financial hardship and becoming a burden to his family. She adjusts his pillow, offers clear explanations about pain management and reassures him that his care will prioritise comfort and dignity. Although this interaction delays other tasks, it alleviates visible distress and strengthens trust between patient and nurse. The benefit is immediate.

This scenario illustrates how compassion alone does not resolve how limited time and attention should be allocated when multiple morally relevant needs compete. Hence the ethical tension. In resource‐constrained environments, where time spent with one patient may reduce attention available to others, how should such a situation be ethically evaluated?

Evaluated through the lens of preference utilitarianism, this scenario reveals both the moral depth of compassionate practice and its inherent tensions. The ethical question it poses is not whether the nurse acted rightly, or if her attentiveness to suffering is morally unambiguous; rather, it is how her decision can be rationally justified when it involves the allocation of a finite resource (time, care or emotional labour) while other patients might be in need of attention.^4^

The first step in a preference‐utilitarian analysis is to identify the full set of preferences at stake. Singer (2011) argues that to think ethically, one's own interests cannot count for more than those of others simply because they are one's own; the moral agent must take into account the interests of all those affected by the decision. In this scenario, the affected parties include the patient himself, whose immediate preferences encompass acknowledgement of his fear, clarity about his care and preservation of his dignity; his family, who await news of his condition; the other patients in the ward, whose needs may be unmet during the nurse's extended presence at the bedside, and the nurse herself, whose preferences include acting professionally and effectively, and sustaining her capacity to respond to suffering across multiple patients, as these preferences ultimately shape the quality and outcomes of care for others. A preference‐utilitarian evaluation cannot privilege any interest in advance; it must weigh competing claims impartially.

The second step concerns the intensity and nature of the preferences involved. A significant complication arises here, however: preferences expressed in conditions of acute distress, incomplete information or existential crisis may be poorly formed or shaped by fear rather than by the patient's considered values (Broome 1991). This is precisely the objection that Brandt's (1979) account of idealised preferences is designed to address. On Brandt's view, what matters morally is not the patient's manifest preferences shaped as they may be by panic, misinformation or the sudden shock of a terminal prognosis, but the preferences he would hold if he were well‐informed and adequately supported. This distinction has a direct implication for nursing practice. The compassionate act of sitting with the patient, listening to his fears and providing clear information about pain management is not merely emotionally responsive. It enables his preferences to become the kind of idealised preferences that carry genuine moral weight. The nurse is not simply responding to suffering; she is helping to stabilise and clarify preferences under conditions of distress, allowing them to be properly formed, expressed and then assessed. This is ethical work.

Once this is recognised, the third step evaluating the relative weight of competing preferences becomes more tractable. Griffin (1986) argues that a sophisticated account of well‐being within the utilitarian framework must extend beyond mere desire‐satisfaction to include components such as deep personal understanding, autonomy and the avoidance of degradation, which is exactly what compassionate care is about. The patient's fear of becoming a burden to his family, his need for clarity about what is to come and his desire that his dying be managed with dignity are not preferences of a trivial or merely immediate kind; they engage the deepest constituents of personal well‐being as Griffin conceives them. The preferences of the ward's other patients, while morally significant, are not comparably urgent at this moment, insofar as their short delay is unlikely to generate comparable levels of distress or harm. This does not license an unlimited allocation of attention to one patient. It identifies the point at which the preference–satisfaction curve shifts—when continuing the nurse's presence at the bedside begins to generate greater unmet preferences elsewhere than it resolves at the bedside.^5^

Additionally, the patient's situation of extreme vulnerability reflects not only the intensity of the preferences involved but also—when viewed through a prioritarian lens—the moral significance of benefiting those who are worst off, compared to the less urgent or less‐harmful‐if‐unmet needs of others. This is not an exception to the principle of impartiality, but rather its considered application, as articulated in the priority view (Parfit 1986).^6^

In this way, the preference‐utilitarian framework enables a structured reflection on how a compassionate response to individual suffering in nursing contexts can be ethically justified, even in scenarios marked by time constraints and concurrent clinical responsibilities. More importantly, it demonstrates how preference utilitarianism can justify the prioritisation of one compassionate obligation over others when competing demands cannot all be met.

Of course, in empirical settings, this framework does not require precise measurement of all preferences or their exact intensities. Rather, it relies on a deliberative process grounded in clinical judgement, experience and sensitivity to patients' expressed and observable needs. Healthcare professionals routinely make reasoned approximations of urgency and significance—distinguishing, for example, between acute distress and more deferrable needs. Here, preference utilitarianism should not be seen as a calculative procedure but as a structured orientation that guides attention towards the most morally salient interests in context.

Consider another hypothetical scenario, which illustrates a conflict of duty between patient safety, timeliness of care and institutional efficiency:

A nurse is stationed at the medication preparation area, responsible for dispensing oral and intravenous drugs for multiple patients. Among the prescriptions is a potent anticoagulant for a patient at high risk of thromboembolic complications. The nurse knows that even a small deviation from the prescribed dose could lead to either insufficient anticoagulation, increasing the risk of clot formation, or excessive anticoagulation, increasing the risk of serious bleeding. By contrast, other routine medications –say, standard vitamin supplements– have relatively minor consequences if small errors occur.

Aware of these differences, the nurse carefully reviews the anticoagulant prescription, checks the patient's recent laboratory results and double‐verifies the dosage with a colleague before adding it to the medication tray. This process takes extra time, which slightly delays the preparation of other patients' medications. However, the nurse judges that the potential harm avoided for the high‐risk patient outweighs the minor inconvenience caused to others. Here, precision becomes compassion when directed intentionally to risk.

Applying the same preference‐utilitarian reasoning, the primary patient's preference is clear: effective anticoagulation that prevents clotting while avoiding excessive bleeding. Other patients have preferences for timely administration of their medications. The institution holds preferences for patient safety, regulatory compliance and accurate documentation. A preference‐utilitarian evaluation requires weighing these interests impartially, without assuming that one patient's needs automatically take precedence over another's.

The relevant preferences include the patient's need for safe and effective anticoagulation, alongside other patients' interests in timely medication administration and institutional priorities such as safety and accuracy. Given the high‐risk nature of anticoagulation, the patient's preference for safe and precise treatment carries significant moral weight, as it depends on technically accurate care.

While other patients' preferences for timely medication administration are morally significant, the potential consequences of a dosing error with a high‐risk drug are disproportionate and could result in severe harm. Given the disproportionate risks associated with dosing errors in high‐risk medications, some require extreme precision. The stakes are not equal. Under these conditions, the preference‐utilitarian approach justifies allocating extra time to the high‐stakes task, since doing so maximises the overall satisfaction of the most morally significant preferences and minimises serious risk.

This case illustrates that compassion in nursing extends beyond bedside interaction. Attentiveness to patient safety, precise execution of professional duties and prioritisation based on the stakes involved all constitute ethically defensible expressions of care. Even when care is indirect, as in medication preparation, sensitivity to the suffering and preferences of patients remains central to morally sound practice: such decisions reflect ongoing prioritisation aimed at reducing harm. The example illustrates how preference‐utilitarian reasoning can guide nurses through conflicts between safety, timeliness and competing patient needs by providing a transparent justification for prioritisation. This guidance matters.

In practice, this utilitarian framing translates into an ethic of action grounded in the alleviation of suffering, respect for dignity and equitable care (Felzmann 2017). This approach demonstrates how rational ethical principles and human sensitivity can be integrated into a coherent framework for decision‐making. While care ethics provides an overarching normative orientation, a compassion‐informed utilitarianism offers a complementary structure for navigating complex clinical realities. This is where compassion becomes accountable.

## 4 Conclusion

Compassion sits at the heart of nursing, yet compassion alone cannot always resolve conflicts between competing patient needs, professional responsibilities and resource constraints. We have argued that contemporary utilitarianism provides a structured framework for navigating such conflicts while remaining attentive to suffering and dignity. Compassion begins with awareness of suffering and culminates in morally responsive action. It restores human connection and sustains dignity within contexts of vulnerability. However, its development and expression cannot rely solely on individual disposition; rather, they depend on organisational and cultural conditions that make compassionate encounters possible, which brings in pressures to navigate competing obligations.

While care ethics highlights the relational and emotional dimensions of moral life, utilitarian reasoning contributes a complementary orientation—one that evaluates actions by their capacity to reduce suffering and promote fairness. These perspectives, when brought into dialogue, need not be viewed as oppositional. Instead, they can form a coherent and context‐sensitive ethical framework. Nevertheless, this integration does not eliminate ethical tensions, particularly in contexts where competing needs cannot be fully reconciled. Instead, it provides a structured framework through which such tensions can be made explicit and critically examined.

A critical part of this framework is the selection of responses that most effectively reduce harm while preserving dignity. Such a process transforms compassion into a deliberative capacity to assess and plan responses to suffering ethically and effectively. It requires judgement.

More specifically, we have argued and illustrated that preference utilitarianism provides accountability, structured prioritisation, fairness and explicit ethical justification under scarcity. Unlike classical formulations, preference utilitarianism directs moral attention to the particular needs, vulnerabilities and circumstances of each person affected, including those least able to articulate or advocate for their own interests. This argument has implications beyond the individual clinical encounter: it places a structural demand on healthcare organisations to ensure that compassionate care is distributed not according to who is most visible or most vocal, but according to where the most significant unmet needs arise.

And while we have presented a *practical framework* for structuring decision‐making in the face of conflicts of duty, the broader moral orientation of nursing remains grounded in overarching theories like care‐based ethics. Utilitarian reasoning, in turn, contributes a complementary practical schema for navigating specific dilemmas, particularly where multiple preferences must be weighed simultaneously.

Our intent has not been to propose a formally self‐contained moral theory, but to reflect the conditions of practice. Clinical decision‐making is inherently pluralistic, often reflecting overlapping influences from care ethics, prioritarian intuitions and, at times, elements of virtue‐based reasoning. In practice, these influences may be seen in the emphasis placed on sustaining therapeutic relationships and responding attentively to individual patient needs, in the tendency to give particular priority to those who are most seriously ill or vulnerable and in appeals to the practical wisdom, compassion and integrity expected of a ‘good clinician’. While the present discussion has focused on bedside decision‐making, where these considerations arise in relation to individual patients, utilitarian forms of reasoning are often applied more explicitly at institutional and public health levels. Rather than eliminating tensions arising from either ethical pluralism or the different scales at which healthcare decisions are made, the framework presented here acknowledges their coexistence as an inherent feature of contemporary clinical reasoning.

This framework should thus be understood as integrating utilitarian evaluation with relational considerations rather than subordinating these to a single theoretical authority. In this sense, in applying a contemporary utilitarian understanding to contexts of compassion, we reaffirm its analytical relevance for modern nursing. Future research may examine how such a framework can be operationalised across diverse clinical settings, particularly in environments characterised by resource scarcity and competing ethical demands. It is in such circumstances that the capacity of utilitarian reasoning to support compassionate care and ethically defensible nursing can be most meaningfully evaluated.

## Funding

The authors have nothing to report.

## Ethics Statement

This research did not involve any participants; therefore, it was not cleared by any ethics committee.

## Conflicts of Interest

The authors declare no conflicts of interest.

## AI and LLM Use

Microsoft Copilot and OpenAI tools may have been used to support sentence refinement and ensure stylistic consistency throughout the manuscript, under the full direction and review of the authors.

## Data Availability

Data sharing not applicable to this article as no datasets were generated or analysed during the current study.

## Footnotes

1 Achieving the most good for the most people may come at the expense of others, who are neglected or even harmed in the process, a problem that frequently arises in public policy.

2 In the late eighteenth century, Jeremy Bentham famously explained: ‘…the question is not whether animals can reason, nor can they talk. The true question is this: Can they suffer?’

3 Negative as in assigning ethical weight to the avoidance of negative states such as suffering, deprivation or harm. Preference as in judging moral rightness by how well actions align with the preferences or interests of individuals.

4 The theoretical foundation for this analysis rests on a core principle articulated by Harsanyi (1977): the only form of utilitarianism consistent with the philosophical principle of preference autonomy is one that determines what is good or bad for an individual solely on the basis of that individual's own desires and preferences, rather than on any external standard of welfare.

5 Not all suffering is morally equivalent to a disvalue to be eliminated. Certain forms of suffering, particularly those bound up with questions of meaning, guilt, spiritual reckoning or personal narrative, can be experienced by patients as constitutive of their situation rather than as an obstacle to well‐being. A preference‐utilitarian framework can accommodate this through Brandt's idealised preferences, even if only to a limited extent: a patient who, once well‐informed and emotionally supported, still chooses to endure a form of suffering as meaningful would have that preference count morally. However, fully articulating the moral significance of meaning‐bearing suffering requires resources beyond the utilitarian framework. In particular, phenomenological accounts treat suffering not as a variable to be minimised, but as a dimension of personhood.

6 This convergence must not be generalised. While, in the present case, prioritising the worst off *coincides* with maximising the reduction of suffering, this alignment is contingent rather than necessary. In triage contexts, for instance, allocating extremely scarce resources to those worst off may fail to produce the greatest overall benefit (i.e., higher chances of recovery) (see Archard and Caplan 2020). The present analysis does not adjudicate between these frameworks; it simply notes that, in the hypothetical scenario under consideration, vulnerability and expected benefit happen to coincide, and that the argument does not depend on prioritarianism as a general principle.
